# Lifetime physical activity and risk of breast cancer

**DOI:** 10.1054/bjoc.2001.2003

**Published:** 2001-09

**Authors:** I-M Lee, N R Cook, K M Rexrode, J E Buring

**Affiliations:** Division Preventive Medicine, Department of Medicine, Brigham and Women's Hospital and Harvard Medical School, 900 Commonwealth Avenue East, Boston, MA, 02215, USA; Department of Epidemiology, Harvard School of Public Health, 677 Huntington Avenue, Boston, MA, 02115, USA; Department of Ambulatory Care and Prevention, Harvard Medical School, 126 Brookline Avenue, Boston, MA, 02215, USA

**Keywords:** breast cancer, epidemiology, exercise, physical activity

## Abstract

We conducted a case–control study of 394 women with breast cancer and 788 control women (91% response) to investigate the association of lifetime physical activity with mainly menopausal breast cancer risk. After controlling for potential confounders, the odds ratios (95% confidence intervals) for increasing quartiles of lifetime physical activity were 1.00 (referent), 0.91 (0.60–1.37), 0.91 (0.60–1.39), and 1.10 (0.73–1.67), respectively;*P*, trend = 0.47. We also separately examined physical activity at ages 12–18, 19–34, 35–49 and ≥50 years; no significant trends were observed in any age group. These data do not support a role of physical activity in preventing breast cancer. © 2001 Cancer Research Campaignhttp://www.bjcancer.com


*British Journal of Cancer* (2001) **85**(7), 962–965
© 2001 Cancer Research Campaign
doi: 10.1054/ bjoc.2001.2003, available online at http://www.idealibrary.com on IDEAL
http://www.bjcancer.com

### Lifetime physical activity and risk of breast cancer

**I-M Lee[1,2], NR Cook[1], KM Rexrode[1] and JE Buring[1,2,3]**

[1]Division Preventive Medicine, Department of Medicine, Brigham and Women's Hospital and Harvard Medical School, 900 Commonwealth Avenue East, Boston, MA 02215, USA; [2]Department of Epidemiology, Harvard School of Public Health, 677 Huntington Avenue, Boston, MA 02115, USA; [3]Department of Ambulatory Care and Prevention, Harvard Medical School, 126 Brookline Avenue, Boston, MA 02215, USA

**Summary** We conducted a case–control study of 394 women with breast cancer and 788 control women (91% response) to investigate the association of lifetime physical activity with mainly menopausal breast cancer risk. After controlling for potential confounders, the odds ratios (95% confidence intervals) for increasing quartiles of lifetime physical activity were 1.00 (referent), 0.91 (0.60–1.37), 0.91 (0.60–1.39), and 1.10 (0.73–1.67), respectively; *P*, trend = 0.47. We also separately examined physical activity at ages 12–18, 19–34, 35–49 and ≥50 years; no significant trends were observed in any age group. These data do not support a role of physical activity in preventing breast cancer. © 2001 Cancer Research Campaign http://www.bjcancer.com

**Keywords**: breast cancer; epidemiology; exercise; physical activity

Breast cancer is the most common cancer occurring among women in Western countries. Unfortunately, almost all established risk factors for this disease (e.g., age at first childbirth or a family history of breast cancer) are not easily altered. One modifiable risk factor that holds promise for prevention is physical activity. Physical activity can modulate levels of female reproductive hormones and influence menstrual characteristics Bernstein et al, 1987). Additionally, active women are more likely to be lean, which is associated with a lower risk of postmenopausal breast cancer (Huang et al, 1997).

However, epidemiologic studies of physical activity and breast cancer risk have yielded inconsistent findings (Gammon et al, 1998a). While several studies have reported lower rates of breast cancer among physically active women than sedentary women (Bernstein et al, 1994; Thune et al, 1997), other studies have not (Dorgan et al, 1994; Rockhill et al, 1998). One reason for this could be the assessment of physical activity at different time periods in a woman's life. The critical time period for physical activity to reduce breast cancer risk may be throughout the life span. Few studies have investigated lifetime physical activity and breast cancer risk. To provide more information, we conducted a case–control study within the Women's Health Study.

### MATERIALS AND METHODS

### Subjects

The Women's Health Study is an ongoing clinical trial testing low-dose aspirin and vitamin E in the primary prevention of cardiovascular disease and cancer among 39 876 apparently healthy female health professionals throughout the United States (Buring and Hennekens, 1992; Rexrode et al, 2000). After an average follow-up of 48 months (February 1998), 411 women developed breast cancer, confirmed by medical records.

At the time we initiated the present case–control study (October 1999), 17 women with breast cancer had died. The remaining 394 women were enrolled as cases in the present study. For each case, we selected 2 controls free of breast cancer at the time the case developed breast cancer, matched to cases on age and date of randomization into the Women's Health Study. The present study was conducted after approval by the institutional review board of Brigham and Women's Hospital. We mailed a questionnaire on physical activity to the 394 cases and 788 controls, and after up to 4 mailings and 2 telephone calls, received questionnaires from 364 cases (92%) and 715 controls (91%).

### Assessment of physical activity

A previously developed physical activity questionnaire was used (Kriska et al, 1988; Pereira et al, 1997), which considers 4 age periods: 12–18, 19–34, 35–49 and ≥ 50 years. For each age period, women were asked to list their leisure-time physical activities. For every activity in each of the 4 age periods, they were asked to report the number of years, months per year, and hours per week the activity was carried out in that age period. This questionnaire, administered 2–3 months apart to postmenopausal women, yielded Spearman correlation coefficients of 0.69–0.85 for the various ages (Kriska et al, 1988).

We estimated the average energy expenditure in MET-hours per week (1 MET-hour = energy expended while sitting quietly for one hour) for each of the 4 age periods (Pereira et al, 1997). We then estimated average lifetime physical activity by calculating a weighted average of the energy expended during the 4 age periods, with the weighting factor being the number of years in the time period (e.g., 7 for the time period 12–18 years). Physical activity was estimated up to the time of entry into the Women's Health Study (i.e., prior to the development of breast cancer for cases).

### Data analyses

We conducted conditional logistic regression to analyse the association between physical activity and risk of breast cancer (Breslow

*Received 17 April 2001*
*Revised 2 July 2001*
*Accepted 2 July 2001*

*Correspondence to:* I-M Lee and Day, 1980). Women were categorized into quartiles of physical activity for each of the 4 age periods, as well as for lifetime physical activity, based on the distribution among controls. We calculated 95% confidence intervals for the estimated relative risks and used 2-tailed tests to calculate *P* values for trend. We also conducted unconditional logistic regression (Breslow and Day, 1980), controlling for the matching factors of age and date of randomization. Because the results from both analyses were similar, we present findings only from the unconditional logistic regression analyses.

We made multivariate analyses, to control for potential confounders, additionally adjusting for randomized treatment assignment, body mass index (continuous variable), alcohol consumption (rarely, 1–3 drinks/month, 1–6/week, ≥ 1/day), age at menarche (≤ 10, single year increments, ≥ 16 years), number of pregnancies lasting ≥ 6 months (none, 1, single unit increments, ≥ 6), age at first pregnancy lasting ≥ 6 months (never pregnant, < 20, 5-year increments, ≥ 35 years), menopausal status (premenopausal, postmenopausal, not sure), ever use of oral contraceptives (no, yes), postmenopausal hormone use (never used, past use, current use), and history of breast cancer diagnosed before age 60 in either mother or sister (no, yes). Information on these variables was obtained from the baseline questionnaire that women completed at entry into the Women's Health Study (i.e., prior to the development of breast cancer in cases).

### RESULTS

Table 1 presents the characteristics of women studied. Cases and controls were matched on age; their mean age at the time of entry into the present study was 56 years. The distribution of physical activity was skewed, with a long tail towards higher values. Among cases, the median value of the average amount of physical activity carried out over the lifetime was 17.5 MET-hours (approximately equivalent to 4–5 hours of brisk walking) per week. Among controls, this was similar, at 17.2 MET-hours per week. When we examined physical activity separately at ages 12–18, 19–34, 35–49 and ≥ 50 years, cases and controls did not differ. Both groups of women were similar with regard to body mass index, postmenopausal status, current use of postmenopausal hormones (among postmenopausal women), and ever use of oral contraceptives. As expected, the following risk factors for breast cancer were present at a higher prevalence among cases: heavy alcohol use, menarche at a younger age, nulliparity and a family history of breast cancer.

We first examined the association between lifetime physical activity, with women categorized into quartiles according to the distribution among controls and risk of breast cancer (Table 2). In analyses that adjusted only for the matching factors of age and date of randomization, we found no association between lifetime physical activity and risk. The most active quartile of women had a similar risk of breast cancer as the least active (odds ratio [OR], 1.04; 95% confidence interval (CI), 0.73–1.48). Additional adjustment for randomized treatment assignment, body mass index, alcohol consumption, menstrual and reproductive characteristics, use of oral contraceptives and postmenopausal hormones, and family history of breast cancer did not materially change the findings (OR, 1.10; 95% CI, 0.73–1.67).

We then examined physical activity separately for the 4 age periods. Physical activity was not clearly predictive of lower risk of breast cancer in any of these age periods (Table 2).

Since previous studies have suggested that the association of physical activity with breast cancer risk may differ among women who are lean or overweight, and among pre- and postmenopausal

**Table 1** Characteristics of cases and controls

| Characteristic | Cases (*n* = 364) | Controls (*n* = 715) |
|---|---|---|
| Mean age (SD), years | 56.0 (7.6) | 56.0 (7.5) |
| Median physical activity, MET-hr/week | | |
| At age 12–18 | 17.0 | 16.9 |
| At age 19–34 | 7.8 | 8.7 |
| At age 35–49 | 15.6 | 16.6 |
| At age ≥ 50 | 21.1 | 22.2 |
| Lifetime[a] | 17.5 | 17.2 |
| Mean body mass index (SD), kg/m$^2$ | 25.1 (4.0) | 25.8 (4.8) |
| Alcohol consumption, % | | |
| Rarely | 40.4 | 42.4 |
| Monthly | 11.0 | 12.2 |
| Weekly | 35.7 | 34.1 |
| Daily | 12.9 | 11.4 |
| Menarche ≥ 14 years, % | 17.3 | 19.2 |
| Nulliparous, % | 14.1 | 8.7 |
| Postmenopausal, % | 63.9 | 64.6 |
| Postmenopausal hormone use[b], % | | |
| Never | 28.5 | 26.8 |
| Past | 12.9 | 15.6 |
| Current | 58.6 | 57.6 |
| Ever used oral contraceptives, % | 63.5 | 64.8 |
| Family history of breast cancer[c], % | 9.2 | 5.0 |

[a]Weighted average of physical activity during ages 12–18, 19–34, 35–49 and ≥ 50 years.
[b]Among postmenopausal women. [c]In mother or sister, with onset < 60 years.

© 2001 Cancer Research Campaign 

**Table 2** Odds ratios (OR) and 95% confidence intervals (CI) of breast cancer, according to physical activity

| Physical activity level | Median, MET-h/wk | No. of cases | No. of controls | 'Crude' OR[a] (95% CI) | Multivariate OR[b] (95% CI) |
|---|---|---|---|---|---|
| Lifetime[c] | | | | | |
| Quartile 1 | 3.0 | 92 | 177 | 1.00 (referent) | 1.00 (referent) |
| Quartile 2 | 11.3 | 88 | 182 | 0.93 (0.65–1.33) | 0.91 (0.60–1.37) |
| Quartile 3 | 23.7 | 89 | 181 | 0.94 (0.66–1.35) | 0.91 (0.60–1.39) |
| Quartile 4 | 52.9 | 95 | 175 | 1.04 (0.73–1.48) | 1.10 (0.73–1.67) |
| | | | | *P*, trend = 0.70 | *P*, trend = 0.47 |
| 12–18 years | | | | | |
| Quartile 1 | 0 | 100 | 169 | 1.00 (referent) | 1.00 (referent) |
| Quartile 2 | 8.8 | 81 | 189 | 0.72 (0.50–1.03) | 0.62 (0.41–0.95) |
| Quartile 3 | 27.5 | 99 | 171 | 0.98 (0.69–1.39) | 0.80 (0.53–1.20) |
| Quartile 4 | 70.5 | 84 | 186 | 0.76 (0.53–1.09) | 0.82 (0.54–1.24) |
| | | | | *P*, trend = 0.37 | *P*, trend = 0.90 |
| 19–34 years | | | | | |
| Quartile 1 | 0 | 99 | 170 | 1.00 (referent) | 1.00 (referent) |
| Quartile 2 | 4.7 | 86 | 184 | 0.80 (0.56–1.14) | 0.79 (0.52–1.20) |
| Quartile 3 | 14.4 | 88 | 182 | 0.82 (0.58–1.18) | 0.82 (0.54–1.24) |
| Quartile 4 | 47.2 | 91 | 179 | 0.87 (0.61–1.24) | 0.84 (0.55–1.27) |
| | | | | *P*, trend = 0.80 | *P*, trend = 0.68 |
| 35–49 years | | | | | |
| Quartile 1 | 0 | 99 | 170 | 1.00 (referent) | 1.00 (referent) |
| Quartile 2 | 9.2 | 89 | 181 | 0.84 (0.59–1.20) | 0.79 (0.52–1.20) |
| Quartile 3 | 23.5 | 84 | 186 | 0.77 (0.54–1.10) | 0.68 (0.45–1.04) |
| Quartile 4 | 57.5 | 92 | 178 | 0.88 (0.62–1.25) | 0.81 (0.53–1.23) |
| | | | | *P*, trend = 0.66 | *P*, trend = 0.50 |
| ≥ 50 years | | | | | |
| Quartile 1 | 2.8 | 72 | 130 | 1.00 (referent) | 1.00 (referent) |
| Quartile 2 | 15.0 | 66 | 137 | 0.88 (0.58–1.32) | 0.72 (0.44–1.20) |
| Quartile 3 | 31.5 | 54 | 148 | 0.66 (0.43–1.01) | 0.56 (0.34–0.93) |
| Quartile 4 | 66.7 | 76 | 127 | 1.08 (0.72–1.62) | 1.04 (0.64–1.70) |
| | | | | *P*, trend = 0.58 | *P*, trend = 0.52 |

[a]From unconditional logistic regression, adjusted for the matching factors of age and date of randomization into the Women's Health Study. [b]Additionally adjusted for randomized treatment, body mass index, alcohol consumption, age at menarche, age at first pregnancy lasting ≥ 6 months, number of pregnancies lasting ≥ 6 months, menopausal status, ever use of oral contraceptives, use of postmenopausal hormones, and family history of breast cancer. [c]Weighted average of physical activity during ages 12–18, 19–34, 35–49 and ≥ 50 years.

women, we examined interactions with body mass index and postmenopausal status. In multivariate analyses, there was no significant interaction with either (*P*, interaction = 0.76 and 0.55, respectively).

### DISCUSSION

These data do not support the hypothesis that physically active women experience lower rates of breast cancer. We did not observe decreased rates of breast cancer among women who were physically active throughout their life from age 12 onwards, or among women who were active at different ages.

Few previous studies have investigated lifetime physical activity in relation to breast cancer risk. Several studies have assessed physical activity at different ages, but did not combine these measures to estimate lifetime physical activity (D'Avanzo et al, 1996; Gammon et al, 1998b; Levi et al, 1999; Morardi et al, 2000). Only 3 studies have integrated assessments of physical activity at different ages in a woman's life to examine the association of lifetime physical activity with breast cancer risk (Bernstein et al, 1994; Carpenter et al, 1999; Verloop et al, 2000). All 3 studies reported lower breast cancer risk among women who were physically active throughout their life from adolescence, with risk reductions of 33% to 58% among the most active women.

Our findings are in contrast with these 3 other studies. It is unclear why the present findings differ. While 2 of the studies examined women who were younger than those in the present study (≤ 40 in Bernstein et al, 1994; and 20–54 years in Verloop et al, 2000), one investigated women of similar age (Carpenter et al, 1999). The questionnaires used to assess lifetime physical activity in these other studies also differed from our questionnaire. Could imprecise assessment of physical activity in our study explain the inconsistency? We believe this is unlikely. The questionnaire we used has good reliability (Kriska et al, 1988) and has face validity. It is extremely difficult to directly validate lifetime physical activity. Based on this instrument, cases and controls had similar levels of physical activity; they also had similar body weight. Both groups of women reported the lowest levels of physical activity between ages 19 and 34 years, and higher levels thereafter. This pattern is consistent with trends in activity in national surveys of US women (US Department of Health and Human Services, 1996). Finally, physical activity levels among women in this study are similar to those among postmenopausal women in another study where physical activity was assessed using the same questionnaire (Kriska et al, 1988).

It is unlikely that the women in the present study were insufficiently active to show effects on breast cancer risk. In 2 studies of lifetime physical activity, women who exercised at least 3.8 hours (Bernstein et al, 1994) or 17.6 MET-hours (approximately equivalent to 4–5 hours of brisk walking) a week (Carpenter et al, 1999) experienced lower breast cancer risk. This level of activity was achieved by about half the women in the present study (median level in controls was 17.2 MET-hours per week).

*© 2001 Cancer Research Campaign*

Lack of statistical power may partly explain the present findings, if the effect of physical activity on breast cancer risk is small to moderate. The 95% confidence interval for the odds ratio of breast cancer among women in the most active quartile of lifetime physical activity in this study, 0.73–1.67 (Table 2), was compatible with up to a 27% reduction in risk. All 3 previous studies of lifetime physical activity observed higher risk reductions (Bernstein et al, 1994; Carpenter et al, 1999; Verloop et al, 2000). However, another large study of physical activity in middle-age and older women reported a risk reduction of only 20% among the most active fifth of women (Rockhill et al, 1999). The present study would not have sufficient statistical power to detect this small difference.

In conclusion, while it is biologically plausible for physically active women to experience lower risk of breast cancer, the present study does not support this hypothesis. Nevertheless, physical activity should still be promoted among women, since it reduces the risk of many chronic diseases, such as coronary heart disease (US Department of Health and Human Services, 1996).

### ACKNOWLEDGEMENTS

This research was supported by grants CA-81611, CA-47988, and HL-43851 from the National Institutes of Health, USA. We would like to acknowledge the crucial contributions of the entire staff of the WHS under the leadership of David Gordon, as well as Mary Breen, Susan Burt, Marilyn Chown, Georgina Friedenberg, Inge Judge, Jean MacFadyean, Geneva McNair, David Potter, Claire Ridge, and Harriet Samuelson. We also would like to acknowledge Drs Wendy Chen and Shumin Zhang of the Endpoints Committee; Colleen Raymond for assistance with data collection; William McMullen and Linda Rose for assistance with data analysis; and Dr Andrea Kriska for the use of the Historical Leisure Activity Questionnaire. Finally, we are grateful to the 39 876 dedicated and conscientious female health professionals who are participating in the WHS.

### REFERENCES

Bernstein L, Ross RK, Lobo RA, Hanisch R, Krailo MD and Henderson BE (1987) The effects of moderate physical activity on menstrual cycle patterns in adolescence: Implications for breast cancer prevention. *Br J Cancer* **55**: 681–685

Bernstein L, Henderson BE, Hanisch R, Sullivan-Halley J and Ross RK (1994) Physical exercise and reduced risk of breast cancer in young women. *J Natl Cancer Inst* **86**: 1403–1408

Breslow NE and Day NE (1980) *Statistical methods in cancer research I: The analysis of case-control studies*. IARC Scientific Pub No. 32. Lyon: International Agency for Research on Cancer

Buring JE and Hennekens CH (1992) The Women's Health Study: Summary of the study design. *J Myocardial Ischemia* **4**: 27–29

Carpenter CL, Ross RK, Paganini-Hill A and Bernstein L (1999) Lifetime physical activity and breast cancer risk among post-menopausal women. *Br J Cancer* **80**: 1852–1858

D'Avanzo B, Nanni O, La Vecchia C, Franceschi S, Negri E, Giacosa A, Conti E, Montella M, Talamini R and Decarli A (1996) Physical activity and breast cancer risk. *Cancer Epidemiol Biomarkers Prev* **5**: 155–160

Dorgan JF, Brown C, Barrett M, Splansky GL, Kreger BE, D'Agostino RB, Albanes D and Schatzkin A (1994) Physical activity and risk of breast cancer in the Framingham Heart Study. *Am J Epidemiol* **139**: 662–669

Gammon MD, John EM and Britton JA (1998a) Recreational and occupational physical activities and risk of breast cancer. *J Natl Cancer Inst* **90**: 100–117

Gammon MD, Schoenberg JB, Britton JA, Kelsey JL, Coates RJ, Brogan D, Potischman N, Swanson CA, Daling JR, Stanford JL and Brinton LA (1998b) Recreational physical activity and breast cancer risk among women under age 45 years. *Am J Epidemiol* **147**: 273–280

Huang Z, Hankinson SE, Colditz GA, Stampfer MJ, Hunter DJ, Manson JE, Hennekens CH, Rosner B, Speizer FE and Willett WC (1997) Dual effects of weight and weight gain on breast cancer risk. *JAMA* **278**: 1407–1411

Kriska AM, Sandler RB, Cauley JA, LaPorte RE, Hom RL and Pambiango G (1988) Assessment of historical physical activity and its relation to adult bone parameters. *Am J Epidemiol* **127**: 1053–1063

Levi F, Pasche C, Lucchini F and La Vecchia C (1999) Occuoational and leisure time physical activity and the risk of breast cancer. *Eur J Cancer* **5**: 775–778

Morardi T, Nyrén O, Zack M, Magnusson C, Persson I and Adami HO (2000) Breast cancer risk and lifetime leisure-time and occupational physical activity (Sweden). *Cancer Causes Control* **11**: 523–531

Pereira MA, Fitzgerald SJ, Gregg EW, Joswiak ML, Ryan WJ, Suminski RR, Utter AC and Zumda JM (1997) Historical leisure activity questionnaire. *Med Sci Sports Exerc* **29** (Suppl): S43–S45

Rexrode KM, Lee I-M, Cook NR, Hennekens CH and Buring JE (2000) Baseline characteristics of participants in the Women's Health Study. *J Women's Health Gend Based Med* **9**: 19–27

Rockhill B, Willett WC, Hunter DJ, Manson JE, Hankinson SE, Spiegelman D and Colditz GA (1998) Physical activity and breast cancer risk in a cohort of young women. *J Natl Cancer Inst* **90**: 1155–1160

Rockhill B, Willett WC, Hunter DJ, Manson JE, Hankinson SE and Colditz GA (1999) A prospective study of recreational physical activity and breast cancer risk. *Arch Intern Med* **159**: 2290–2296

Thune I, Brenn T, Lund E and Gaard M (1997) Physical activity and the risk of breast cancer. *N Engl J Med* **336**: 1269–1275

US Department of Health and Human Services (1996) *Physical activity and health: A report of the Surgeon General*. Atlanta, GA: US Department of Health and Human Services, Centers for Disease Control and Prevention, National Center for Chronic Disease Prevention and Health Promotion

Verloop J, Rookus MA, van der Kooy K and van Leeuwen FE (2000) Physical activity and breast cancer risk in women aged 20–54 year. *J Natl Cancer Inst* **92**: 128–135

*© 2001 Cancer Research Campaign*
